# Assessing biological activities of polyphenols in the context of 4R – challenges of a ubiquitous compound class with complex modes of action

**DOI:** 10.3389/fphar.2026.1817679

**Published:** 2026-04-15

**Authors:** Verena Spiegler

**Affiliations:** Institute of Pharmaceutical Biology and Phytochemistry, University of Münster, Münster, Germany

**Keywords:** 4R, bioavailability, *in vitro* assay, pleiotropic effects, polyphenol

## Abstract

Polyphenols are widespread in plants used for nutritional and medicinal purposes, and have been associated with multiple beneficial effects on health. From a drug development perspective, however, many polyphenols have been disapproved as lead structures and designated as nuisance compounds. Nevertheless, research on health promoting effects of polyphenols has continuously increased over the past decade. Given their ubiquitous presence in plants and their occurrence as mixtures, an application in form of herbal preparations could be more advantageous than the administration of single compounds with numerous, but often weak effects of unclear relevance. Currently, the most powerful technique attempting to capture the entirety of pleiotropic activities of herbal medicines is network pharmacology. However, the major challenges and limitations related to assessing the pharmacological effects of polyphenols, such as unspecific interactions in assays, pharmacokinetic properties and synergisms among extract ingredients largely remain, and can lead to uncertainties regarding the validity of the data input. Especially in light of the 4R principle for animal experimentation, attention to planning meaningful experiments continues to be of high importance when studying bioactivities of polyphenols. This mini review therefore revisits characteristics and challenges associated with pharmacological investigations of polyphenolic natural products to discuss them in the context of 4R.

## Introduction

Around fifteen to 20 years ago, recurrent promiscuous activities of certain compound classes and molecule substructures in biological *in vitro* assays became so apparent that alerts were published to raise the awareness of researchers conducting biological screenings for active compounds (Baell, 2010; Baell and Walters, 2014; Feng et al., 2005). The term PAINS, pan assay interference compounds, was shaped to summarize these bad acting molecules (Baell, 2010). Typically, PAINS interfere with protein- or cell-based assays through their chemical reactivity which is often associated with certain functional groups, such as quinones or catechols prone to covalent reactions with proteins, complexation of metal ions or redox cycling Further modes of assay interference include compound fluorescence or colorization, aggregation, instabilities or reactions with assay reagents (Baell, 2010; Baell and Walters, 2014; Dahlin et al., 2021; Thorne et al., 2010). Based on these false positive screening hits, such compounds are then claimed to be lead structures, although the chances for successful further optimization are expected to be low (Baell, 2010; Baell and Walters, 2014). Certainly, assay-interference is not a phenomenon exclusive to the field of medicinal chemistry and synthetic compounds. Shortly after introducing the PAINS to a broad scientific audience (Baell and Walters, 2014), concerns were extended to natural products possessing suspicious substructures likely to show unfavorable reactivities, but also perturbation of membranes, during evaluation in biological assays (Baell, 2016; Glaser and Holzgrabe, 2016; Ingólfsson et al., 2014). In the same context, frequent hitters have been identified among compounds of natural origin and termed invalid metabolic panaceas (IMPs). Of note, IMPs not only comprise promiscuously active natural products, including some with PAINS motifs, many of them represent secondary metabolites that are ubiquitously present in plant species (Bisson et al., 2016). Already with the definition of PAINS, the author called for a more cautious interpretation of screening results and also addressed the responsibility of journals and their reviewers (Baell, 2010) to avoid a propagation of irrelevant data through continuous reporting. The current review aims at summarizing the challenges and developments concerning studies on herbal medicines with focus on polyphenols due to their high representation among top-ranked IMPs and occurrence of PAINS motifs (Bisson et al., 2016). In addition to wasting laboratory resources and time, invalid *in vitro* (and *in silico*) results may also be a motivation for subsequent studies in animals. Implications of assaying such natural products *in vivo* and the compliance with the 4R principle will therefore particularly be addressed.

### Polyphenols possess multi-modal activities and are widespread in plants and multicomponent herbal medicines

In general, polyphenols comprise a structurally diverse class of plant secondary metabolites, ranging from small molecules to polymers. A common feature is the presence of more than one phenolic ring that biosynthetically originated from the shikimate and/or the polyketide pathway (Quideau et al., 2011). Looking at the number of publications reporting *in vitro* assay results, phenolic compounds still seem to continuously and increasingly attract the interest of researchers. Particularly the determination of pharmacological targets for flavonoids has gained increasing attention in the past years (Wang et al., 2023), suggesting that the discussions of their promiscuous activities did not lead to an obvious decline in research efforts. Certainly, one reason for this observation is the widespread occurrence of phenolics, especially flavonoids, as important metabolites of (medicinal) plants (Bisson et al., 2016; Treutter, 2005; Yonekura-Sakakibara et al., 2019), together with the continuing interest in understanding their beneficial effects on health. Plant extracts employed for medicinal purposes usually represent complex mixtures of secondary metabolites (Efferth and Koch, 2011; Gertsch, 2011; Wink, 2008). Attempts to rationalize the medicinal use of plant extracts, apart from clinical trials include the correlation of metabolites identified in extracts with biological activities, which are usually assessed in cell-based assays *in vitro*. This has long been approached by performing bioassay-guided fractionations aiming to trace the activity of an extract ideally to single components. The identification of an active compound in a mixture follows the idea that distinct metabolites are responsible for distinct biological effects, supposedly by interacting with one or few defined targets. However, frequently enough, this approach is unsuccessful due to insufficient amounts of bioactives for isolation, degradation of the compounds of interest during purification and the disappearance of activity during fractionation, which can be attributed to synergistic effects among extract constituents (Dettweiler et al., 2020; Nothias et al., 2018). To better account for the multicomponent character of plant extracts and to avoid common pitfalls of bioassay-guided fractionations, especially in the past decade, improved methods were established allowing to assess complex mixtures more efficiently. On the one hand, this has greatly facilitated dereplication of secondary metabolites to rapidly detect known compounds and to avoid re-isolation of metabolites (Fox Ramos et al., 2019; Hubert et al., 2017; Meunier et al., 2024). Particularly the GNPS platform that provides community shared MS/MS spectra (Wang et al., 2016) has gained great popularity for dereplication and molecular networking. In addition, ^13^C NMR- and HSQC-based workflows have been established as alternatives to mass spectrometry (Bruguière et al., 2020; Stienstra et al., 2025). On the other hand, biochemometric approaches, including bioactivity-based molecular networks, enabled a rapid association of metabolites with biological activities (Fox Ramos et al., 2019; Meunier et al., 2024; Naman et al., 2017; Nothias et al., 2018; Olivon et al., 2017; Wang et al., 2016). Moreover, metabolomics can also aid in revealing synergies or antagonisms among extract metabolites (Caesar and Cech, 2019) as well as in pharmacokinetic profiling of plant extracts (Seo et al., 2022). A main goal of bioactivity-based molecular networking is to identify bioactive constituents of an extract at an early stage to avoid repeated isolation of the same known bioactives (Naman et al., 2017; Nothias et al., 2018). Typically, bioactivity data are obtained for the identical samples that are chemically analyzed, resulting in a coherent experimental set of analytical and pharmacological data for a given specimen. Still, biochemometric models rely on the quality of the underlying biological assays (Caesar and Cech, 2019) and the possibility that promiscuously acting compounds are spotted as bioactives remains. However, a major aim of biochemometric approaches is the prioritization of molecules to be isolated, usually new natural products or previously unknown actives. Hence, the possibility to waste resources on the purification of known frequent hitters still exists, but is reduced compared to other workflows if researchers are sensitized to the problem. Integration of multiple bioassay results into molecular networks can even increase the chance to detect promiscuous activities (Olivon et al., 2017). Although available reference datasets for dereplication are expanding due to efforts of the community in data sharing, in line with the FAIR principles (Wilkinson et al., 2016), machine learning algorithms have been developed, permitting dereplication based on *in silico* fragmentation (Fox Ramos et al., 2019; Saldívar-González et al., 2022) or mass spectrometry-independent techniques (Saldívar-González et al., 2022). Moreover, machine learning offers unprecedented opportunities to predict biological activities, but also further parameters such as pharmacokinetic properties of natural products (Chigozie et al., 2025; Othman et al., 2025; Saldívar-González et al., 2022; Xue et al., 2022; Zhang et al., 2021). In any case, as the success of machine learning predictions depends on data from biological assays (Xue et al., 2022; Zhang et al., 2021), the reliability of the dataset integrated into the study represents a crucial factor for the success of AI-based identification of bioactives (Chigozie et al., 2025; Zhang et al., 2021). Current limitations that have been specified for natural product datasets mainly include incomplete structural information, inaccessibilities, inappropriate training sets, an overrepresentation of positive instances, lack of structural information regarding the target, inconsistent or non-standardized information on bioactivities (Chigozie et al., 2025; Rodrigues, 2019; Xue et al., 2022; Zhang et al., 2021) and finally, false positive hits in assays (Rodrigues, 2019). Conversely, as machine learning advances, it will provide increasingly reliable possibilities to identify and exclude nuisance compounds from further research efforts (Reker et al., 2019; Rodrigues, 2022).

Plants in particular, however, do not rely on single molecules for defense, but on mixtures, that not only exert pleiotropic effects (Schmidt et al., 2007) due to the sum of different targets addressed by different molecules, but also by metabolites targeting multiple protein structures with low affinity (Efferth and Koch, 2011; Gertsch, 2011; Wink, 2008). Together with synergistic (or antagonistic) effects among the metabolites contained in an extract mixture, multi-target activities have therefore been considered a characteristic as well as an advantage of phytotherapy (Efferth et al., 2017; Efferth and Koch, 2011; Gertsch, 2011; Schmidt et al., 2007; Wagner and Ulrich-Merzenich, 2009; Wink, 2008). However, with respect to estimating biological activities in *in vitro* assay systems, multicomponent mixtures pose by far more challenges than those already observed for single compounds. Even more, if traditional plant-based medications consist of preparations from more than one plant species. One approach that attempts to comprehensively capture the multi-modal character of phytomedicines is network pharmacology. Initially, it challenged the paradigm of one disease–one target–one drug (Hopkins, 2008), unrelated to considerations for phytotherapy. However, it rapidly evolved as a tool to achieve an improved understanding of pleiotropic effects elicited by plant extracts. Network pharmacology has gained particular interest for studying complex multi-component mixtures used in Traditional Chinese Medicine (TCM), but it is certainly not restricted to it. Research articles and reviews applying network pharmacology to various preparations and disease models, as well as discussing its potential in TCM research are so numerous that summarizing all developments in this field would go beyond the scope of this perspective. Briefly, network pharmacology aims at evaluating effects of drugs on disease-related pathways and networks of interacting proteins and targets (Hopkins, 2008; Panossian, 2025). Main goals of its application in TCM include the determination of active ingredients, an insight into the mechanisms of action, interactions between components, biomarker identification, the prediction of drug efficacy or toxicities, and the identification of new indications for drug repurposing (Wang et al., 2021; Wang Y. et al., 2024; Yang et al., 2025; Zhai et al., 2025; Zhang et al., 2013). Several recent reviews provide detailed information on methodologies, the selection of appropriate databases as well as strengths and limitations of network pharmacology (Lee et al., 2025; Panossian, 2025; Wang Y. et al., 2024; Yang et al., 2025; Zhai et al., 2025). Common workflows can roughly be summarized as follows: In one of the first steps, metabolomic analyses are conducted to collect information regarding the phytochemical composition of the extracts. Detected ingredients are then mapped to targets, either based on curated databases containing information mainly from *in vitro* experiments, or based on *in silico* prediction (Lee et al., 2025; Wang Y. et al., 2024; Yang et al., 2025). Unrelated to any specific methodology or medicinal preparation, the risk of mining invalid biological data when handling large datasets has previously been pointed out in the context of promiscuously acting compounds (Bisson et al., 2016; Newman, 2021; Rodrigues, 2019). And similarly, the potential of identifying unsuitable compounds through *in silico* and *in vitro* prediction has recently been highlighted in the context of network pharmacology (Panossian, 2025; Yang et al., 2025). In some cases, the method is employed to predict key compounds deemed to significantly contribute to the overall activity of a preparation (Noor et al., 2022; Zhang et al., 2016). Again, as exemplified by recent reports, polyphenols remain strongly represented (Noor et al., 2022; Wang X. et al., 2024; Xu et al., 2026; Yang et al., 2025). At the same time, some polyphenols frequently identified in TCM preparations have been associated with a high risk to give false positive assay readouts by colloidal aggregation (Duan et al., 2015). Whether their frequent appearance in network pharmacology may be explained by their ubiquitous occurrence in plant materials or the long record of reported activities that may affect the prediction of targets based on collected references for these compounds remains to be clarified. However, Reker et al. comprehensively illustrated the impact of erroneous assumptions concerning the binding to proteins for the accuracy of results obtained from network pharmacology. They also estimated that a strikingly high proportion of ChEMBL entries for bioactivities of small colloidally aggregating molecules may have derived from false positive assay results (Reker et al., 2019), which in summary underlines the importance of database curation and the impact rampant dissemination of invalid bioactivity data may have for downstream research efforts.

### Pharmacokinetics remain a key issue

A key determinant for a compound to exert any pharmacological effect *in vivo* is its bioavailability and concentration at the target site. Therefore, considerations of ADME properties are usually included in network analyses, at least as *in silico* predictions (Noor et al., 2022; Wang Y. et al., 2024; Yang et al., 2025; Zhai et al., 2025), although one limiting factor is the strong focus on oral bioavailability (Yang et al., 2025). In general, the fundamental need to take pharmacokinetics into account when studying biological activities of natural products and herbal medicines has repeatedly been stressed (Butterweck and Nahrstedt, 2012; Gertsch, 2011; Wang et al., 2023). Comprehensive compilations of the numerous data concerning the pharmacokinetic properties of different polyphenols in humans have been provided (Del Rio et al., 2013; Di Pede et al., 2023; Manach et al., 2004; Manach et al., 2005; Rubio et al., 2014). Given the broad distribution of polyphenols in foods, a large body of knowledge on their bioavailability stems from studies on dietary sources rather than herbal medicines that might contain different compositions and concentrations of polyphenols. Nevertheless, the general properties are independent of the source and point out the challenges associated with the request to better integrate pharmacokinetic information into pharmacologic experiments: The absorption of polyphenols in general is highly variable and depends on the exact compound structure (Del Rio et al., 2013; Manach et al., 2005; Rubio et al., 2014). Flavonoids, for example, commonly occur as glycosides or other modified derivatives rather than in form of their aglycon (Veitch and Grayer, 2008; Yonekura-Sakakibara et al., 2019). The glycosylation pattern as well as the matrix were found to greatly influence their absorption in the small intestine (Borges et al., 2022; Del Rio et al., 2013; Liu L. et al., 2025; Manach et al., 2005; Rubio et al., 2014). In addition, substantial interindividual variation has been reported which was especially observed for the absorption of metabolites formed by the colon microbiota (Favari et al., 2024) that add to or even exceed the effects of polyphenols (Luca et al., 2020). A prominent example are the urolithins that result from the microbial transformation of ellagitannins and can reach significant plasma concentrations (Cerdá et al., 2004). Within a recent clinical trial, for example, the concentration of these metabolites after intake of raspberry drinks were correlated with improvements in endothelial function (Istas et al., 2018). Except for epigallocatechin gallate that appeared unchanged in plasma samples (Clifford et al., 2013), most polyphenols are rapidly conjugated to glucuronides, sulfates or methyl esters during absorption from the small intestine or in the liver, and cannot be found in non-conjugated forms in plasma (Del Rio et al., 2013; Kroon et al., 2004; Manach et al., 2004; Manach et al., 2005; Rubio et al., 2014; Scalbert et al., 2002). After absorption, metabolites derived from the gut microbiota similarly undergo phase II metabolism (Di Pede et al., 2023). Concerning unspecific chemical reactivities of polyphenols as observed in *in vitro* assays, the extensive metabolization, particularly the *O*-methylation of catechol moieties, has been associated with a lower risk of toxicity due to decreased redox cycling (Kroon et al., 2004; Scalbert et al., 2002), while the resulting conjugates can retain antioxidant functions (Gebicki and Nauser, 2021; Kroon et al., 2004). Therefore, the consideration of physiologic conjugates at realistic concentrations has repeatedly been called for when planning *in vitro* experiments (Borges et al., 2022; Kroon et al., 2004; Manach et al., 2004). It is to mention that assaying polyphenol metabolites does not rule out unspecific or promiscuous effects. Similar to interactions of flavonoids with biological membranes (Hendrich, 2006), quercetin and its major phase II metabolites have been found to penetrate into lipid bilayers, however, to a different extent, depending on the substituent (Košinová et al., 2012). Membrane interactions of a compound do not *per se* allow any conclusion on its efficacy. They have, for example, been associated with beneficial effects by polyphenols and their metabolites on blood pressure (Reis et al., 2024).

Further, polyphenols are extensively bound to plasma proteins (Manach et al., 2004), but much less is known regarding the bioavailability of metabolites in tissues. In general, it does not correlate with plasma concentrations and the uptake highly varies with the type pf polyphenol, its metabolites and the respective tissue (Manach et al., 2004). Resveratrol, for example, has been found to accumulate in kidneys, liver or intestinal tissues in rodents (Walle, 2011), whereas some polyphenols and low molecular weight metabolites from the colonic microbiome may cross the blood-brain barrier (Carecho et al., 2021). Moreover, the possibility of metabolite deconjugation has been observed at the cellular level (Manach et al., 2004) and this process may be influenced by disease specific alterations, affecting the selectivity of polyphenols depending on the respective conditions (Sheridan and Spelman, 2022). In general, the low bioavailability of polyphenols is a topic of frequent discussion, especially in studies assessing free, non-conjugated polyphenols *in vitro*. Suggestions to enhance their bioavailability by improved formulations or other measures occasionally create the impression that such efforts could turn polyphenolics with otherwise moderate or negative clinical evidence into silver bullets. Undoubtedly, improving the pharmacokinetic properties of compounds can lead to success, but the selectivity and potential adverse effects need to be considered. Within a critical perspective regarding the utility of curcumin as a lead compound, the authors also commented on potential toxicological side effects associated with an increased bioavailability (Nelson et al., 2017). Indeed, several case reports of patients suffering liver failure after consumption of curcumin in combination with bioavailability enhancers, such as piperine, have accumulated (Lombardi et al., 2021; Smati et al., 2023). While the mechanisms of toxicity are yet unknown and other factors could not be entirely ruled out, further research on toxicologic risks arising from curcumin preparations with increased bioavailability has been demanded (BfR, German Federal Institute for Risk Assessment, 2021).

Similar to matrix effects in food sources, the presence or absence of other plant secondary metabolites and their influence on metabolizing enzymes (Rubio et al., 2014) can greatly affect the pharmacokinetic properties of certain extract ingredients (Efferth and Koch, 2011; Wagner and Ulrich-Merzenich, 2009). An impressive example is the dependency of antidepressant effects by St. John’s wort (*Hypericum perforatum*) extracts in rats on a sufficient concentration of rutin (Nölder and Schötz, 2002). Thus, considering all of these factors and the utilization of one or more non-standardized plant preparations with variable phytochemical composition in most preclinical investigations (Gertsch, 2011; Wang et al., 2023), the setup of any *in vitro* assay attempting to reflect *in vivo* conditions seems to be an unattainably complex endeavour. And this is even more the case for *in silico* methods, such as molecular docking, if binding to distinct target structures is assumed, although neither the actual ligand (the natural product itself or a metabolite?) is ascertained, nor its ability to reach the predicted target at all. On the other hand, while evidence from human trials is increasing, databases compiling meaningful information for polyphenols, such as the Phenol-Explorer for polyphenolic food ingredients (Neveu et al., 2010; Rothwell et al., 2012; Rothwell et al., 2013), could provide a good base for future AI-supported research on polyphenols (Zheng et al., 2024). A metabolomic approach integrating patient derived pharmacokinetic information for herbal medicines, the Poly-PK strategy, has already been introduced by Jia et al. (2015).

### The balance between meaningful studies of polyphenols and the 4R principle

Due to the challenges associated with *in vitro* and *in silico* studies of polyphenols outlined above, *in vivo* experiments to validate *in silico* and *in vitro* findings have repeatedly been called for (Butterweck and Nahrstedt, 2012; Panossian, 2025; Yang et al., 2025). While this is scientifically plausible, it appears to conflict the 4R principle for animal experiments.

Originally, three principles were proposed by Russell and Burch in 1959 in order to improve the quality of animal studies through a humane treatment of experimental animals (Russell and Burch, 1959): Replacement, Reduction and Refinement. Replacement refers to the substitution of sentient animals by insentient materials. Reduction aims at gaining a reliable result using a reduced number of animals, whereas refinement means to diminish the extent to which animals are exposed to inhumane treatments (Russell and Burch, 1959; Tannenbaum and Bennett, 2015). Later, responsibility was added as the fourth R as part of the White Paper on Animal Research issued by the Max Planck Society to address the commitment of researchers to enhance the welfare of laboratory animals (Blakemore et al., 2016). At first glance, demands for *in vivo* studies to generate valid research results, especially for compound classes like polyphenols and their multi-modal activities, seem to counteract the principles of 4R. Particularly, in view of limitations in predictivity and reliability of data obtained from animal experiments for humans (van Norman, 2019), which has also been stated in the context of phytomedical research (Yang et al., 2025). In the past years, the development of organoids, 3D culture systems sharing high similarity with human organs, has opened new avenues to access human specific processes in biomedical studies that cannot be modelled in animals (Kim et al., 2020). Organoids have also been employed to investigate bioactivities of natural products (Cong et al., 2025; Liu S. et al., 2025). However, given the complexity of polyphenol actions in humans described above, none of the current *in vitro* models (or insentient materials) can be considered fully adequate to afford valid results for these compounds (Wang et al., 2023), and most likely for the majority of herbal medicines. Still, following the principle of Replacement could be a motivation to turn to research questions not requiring animals to yield the desired outcome (Lauwereyns et al., 2024). In case of herbal medicines in general, we are in the fortunate situation that they are integrated into the different healthcare systems worldwide. Unlike clinical trials for the approval of new active pharmaceutical ingredients, patients are treated with various kinds of herbal medicines on a daily base. Thus, clinical data generally exist, ranging from randomized placebo-controlled trials to case reports and also include pharmacokinetic investigations. This extends to intervention studies for botanicals or single natural products applied as dietary supplements and to health promoting effects due to long term consumption of certain foods. Certainly, clinical data are not available for many compounds or preparations and obtaining them is usually associated with ethical and economical impediments. However, more frequently integrating existing data, e.g., regarding the bioavailability of polyphenols, into cell-based experiments as in the example for tea flavonoids (Wiseman et al., 2001) could be decisive for further experiments. If a compound, irrespective of promiscuous activities, does not exert an effect at a realistic concentration estimated based on data from patients, there is never a need nor a justification to perform an animal experiment.

A closer collaboration among researchers from clinical and non-clinical disciplines could also be regarded as a Reduction measure in the broader sense (Lauwereyns et al., 2024). More generally, two points can be addressed in the context of research with polyphenols related to the Reduction principle if it was understood as reducing the number of animal experiments, in addition to the number of animals per study as per the initial definition (Lauwereyns et al., 2024; Russell and Burch, 1959). First, if a hypothesis is inferred from *in vitro* data or *in silico* predictions that are obviously unrealistic according to current knowledge, this should not provide an inspiration to plan further animal experiments. The second point again addresses pharmacokinetic aspects and is already closely linked to Refinement: Butterweck and Nahrstedt pointed to frequent mismatches between the route of administration for extracts and natural products in traditional medicine and their administration in rodents (Butterweck and Nahrstedt, 2012). Intraperitoneal injections are simple to perform in practice, but several issues, such as an altered absorption, the formation of metabolites by the microflora and pharmacokinetic interactions between extract constituents limit the significance of *in vivo* experiments employing a route of administration irrelevant to the use in humans (Butterweck and Nahrstedt, 2012). Given that polyphenols and herbal medicines are regularly consumed or administered orally, the large impact of metabolites on the bioactivity is not adequately accounted for when choosing a peritoneal administration. In addition, the authors address another problem related to Refinement, which concerns the potentially negative impact on the experimental results due to irritations and stress by the injections (Butterweck and Nahrstedt, 2012). Not only is this invasive route of administration unnecessary and harmful, it also compromises the validity of the result and, therefore, stands in stark contrast to the Refinement principle (Lauwereyns et al., 2024; Russell and Burch, 1959). Despite government commitments and breakthroughs in the development of New Approach Methodologies, certain research questions currently still rely on animal experiments (Burki, 2026). To reduce suffering and distress, invertebrate animals like the fruit fly (*Drosophila melanogaster*) or the roundworm *Caenorhabditis elegans*, as well as distantly related vertebrates such as zebrafish (*Danio rerio*) have been proposed as model organisms to replace mammals and contribute to Refinement (Lauwereyns et al., 2024; Poh and Stanslas, 2024). While organoids can be considered superior in reflecting the conditions in humans, they cannot mimic the physiologic complexity of an entire organism (Kim et al., 2020). Therefore, such models can bear clear advantages over cell cultures and all of the species mentioned have been used to study biological effects of plant extracts and natural products (Sheng et al., 2025; West and Crawford, 2016; Wink, 2022), including polyphenols (Carregosa et al., 2021; Chen et al., 2024; Jimenez-Del-Rio and Velez-Pardo, 2015; Liu et al., 2021; Mhalhel et al., 2023; Montalbano et al., 2021; Pallauf et al., 2017). Most frequently, they serve as models for neurodegenerative diseases or longevity. *Caenorhabditis elegans* and *D. melanogaster* share the advantages of short lifespans, easy maintenance under laboratory conditions and comprehensive genomic annotations with homologs in humans (Pallauf et al., 2017; Sheng et al., 2025), whereas zebrafish additionally possess organs and tissues equivalent to humans, making this model particularly useful to estimate drug efficacies (Patton et al., 2021). Further, *C. elegans* has been proposed as a model organism to investigate the tissue distribution of polyphenols (Santos et al., 2014). Examples of using this roundworm for bioavailability studies are scarce, but a similarly extensive conjugation to sulfates and glucuronides as in humans has been observed for a selection of flavonoids administered to *C. elegans* (Grünz et al., 2012). However, at least with respect to experiments on lifespan extension, the relevance for humans of findings observed in *C. elegans* and *D. melanogaster* has been questioned (Burdusel et al., 2024). Moreover, “lower” or more distantly related animals remain to be living organisms and their sentience is beyond our knowledge. In particular zebrafish as a vertebrate animal does not receive the same attention as mammalian organisms regarding protection and wellbeing, despite respective guidelines for fish (Canedo et al., 2022). 10Rs have therefore been recommended for research with zebrafish, extending the 4Rs by further five scientific principles. One of them concerns the reporting (Canedo et al., 2022), which is also an integral aspect of the ARRIVE guidelines that provide detailed recommendations for reporting animal research (Du Percie Sert et al., 2020) and should be attended to when planning and publishing experiments with animals, using polyphenols or any other class of compounds. Further, registration of the experiments, robustness of the results and a diligent evaluation of the relevance and suitability of the animal model have been called for (Canedo et al., 2022). Similar considerations have not (yet) been devised for *C. elegans*, *D. melanogaster* or other model organisms. Nevertheless, because all points raised above regarding *in vitro* assays with polyphenols, likewise affect invertebrate animal models, the translatability of the experiments for the conditions in humans needs to be evaluated with the same care as for cell culture or rodent models.

## Conclusion

In summary, pharmacological research on polyphenols remains a challenge. It is important to note that the aim of the current review is not to judge on methodologies nor on any efficacy or plausibility of applications for this class of natural products, but to highlight their characteristic properties, the difficulties to adequately incorporate them into *in vitro* assays and the implications for animal experiments. Although, from a drug discovery perspective, blind adherence to PAINS filters is not (and has not been) advised (Baell, 2016; Baell and Nissink, 2018; Choo and Chai, 2022) and the concept of GAINS (give attention to limitations in assays) has been put forward, polyphenols certainly still disqualify as classic lead structures (Choo and Chai, 2022). Does that also imply that research on polyphenols inevitably leads to irrelevant results or can the idea of GAINS be transferred to phytotherapy and natural product pharmacology? Certainly, polyphenol research can be meaningful, but some points need clarification. It should simply be accepted that a majority of *in vitro* activities is caused by unspecific chemical reactions, foremost membrane perturbations that affect cell signaling pathways (Choo and Chai, 2022; Ingólfsson et al., 2014). Further efforts to develop single polyphenolics into potent medications should therefore be regarded with sufficient scepticism. In turn, it would similarly be wrong and oversimplified to conclude that polyphenols in no case can be useful medications. Tannins, for example, combine all properties to qualify them as promiscuous nuisance compounds. Still, sinecatechins or crofelemer, for example, represent the active components of approved drugs with demonstrated clinical efficacy. Moreover, as shown by a growing number of studies, shifting the focus for low molecular weight polyphenols away from the parent compounds to the respective metabolites holds promise for exciting new insights into their bioactivities. The central question, not only relevant to polyphenols, therefore remains, whether the outcome of a pharmacological assay can be translated to therapeutic effects in humans (Butterweck and Nahrstedt, 2012). Another point is that “promiscuous activity” can be negatively regarded as a false positive screening hit or optimistically interpreted as conveying multi-target effects. As again exemplified by tannins and their unspecific protein binding properties, a specific target is not required for the efficacy of a treatment. It might therefore in some cases be more straightforward to recognize membrane interactions as the actual mechanism of polyphenol action, instead of trying to define specific proteins as binding partners with questionable validity. This does not contradict the idea of multicomponent mixtures exerting concerted activities on signaling pathways (which should ideally be confirmed *in vivo*). Moreover, unspecific mechanisms may hold promise with respect to addressing diseases where resistances are increasingly emerging. In any case, a realistic estimation of bioavailabilities remains to be both, essential to evaluate the relevance of assay results as well as a continuous challenge in phytotherapy research, including polyphenols. Given the limitations of *in vitro* tests, the need for *in vivo* animal studies continues. Nevertheless, careful considerations with respect to the principles of 4R are required. Even more so, if invalid *in vitro* results and an uncritically optimistic appraisal of alleged polyphenol activities forms the base and motivation of these studies. Unfortunately, the ever-growing number of articles reporting bioactivities for polyphenolics becomes hardly manageable and the amount only of review articles seems to outnumber the publications of clinical data by far. A recent systematic review summarized the clinical evidence for various effects of flavonoids, stating that the level of evidence is frequently too low to allow firm conclusions on their efficacy (Wang et al., 2023). Paying closer attention to the relevance of *in vitro* data as well as to limitations and negative clinical outcomes would therefore be an advice to counteract overenthusiastic claims. Although the problems that occur when assaying polyphenols have generally been acknowledged, it still seems indicated to reinforce previous appeals concerning scientific practices in dealing with botanicals (Butterweck and Nahrstedt, 2012; Gertsch, 2009; 2011; Heinrich et al., 2020). In line with the introduction of GAINS, the intention should not be to discourage efforts to work with polyphenols or herbal medicines, but to direct the endeavors towards gaining meaningful insights for potential applications.
